# Impact of morning stiffness on working behaviour and performance in people with rheumatoid arthritis

**DOI:** 10.1007/s00296-014-3040-0

**Published:** 2014-05-29

**Authors:** Kalle Mattila, Frank Buttgereit, Risto Tuominen

**Affiliations:** 1 Department of Public Health, University of Turku, Lemminkäisenkatu 1, 20014 Turku, Finland; 2 Department of Rheumatology and Clinical Immunology, Charité University Medicine, Charitéplatz 1, 10117 Berlin, Germany; 3 Primary Health Care Unit, Hospital District of Southwest Finland, Turku, Finland

**Keywords:** Rheumatoid arthritis, Morning stiffness, Work performance, Early retirement

## Abstract

Work disability remains a considerable problem for many patients with rheumatoid arthritis (RA). Morning stiffness is a symptom of RA associated with early retirement from work and with impaired functional ability. We aimed to explore the patient’s perception of the impact of morning stiffness on the working life of patients with RA. A survey was conducted in 11 European countries. Patients of working age, with RA for ≥6 months and morning stiffness ≥3 mornings a week, were interviewed by telephone using a structured questionnaire. Responses were assessed in the total sample and in subgroups defined by severity and duration of morning stiffness and by country. A total of 1,061 respondents completed the survey, 534 were working, 224 were retired and the rest were, i.e. homemakers and unemployed. Among the 534 working respondents, RA-related morning stiffness affected work performance (47 %), resulted in late arrival at work (33 %) and required sick leave in the past month (15 %). Of the 224 retired respondents, 159 (71 %) stopped working earlier than their expected retirement age, with 64 % giving RA-related morning stiffness as a reason. There was a differential impact of increasing severity and increasing duration of morning stiffness on the various parameters studied. There were notable inter-country differences in the impact of RA-related morning stiffness on ability to work and on retirement. This large survey showed that from the patient’s perspective, morning stiffness reduces the ability to work in patients with RA and contributes to early retirement.

## Introduction

The introduction of biological therapies may have had some positive effects on work participation in patients with rheumatoid arthritis (RA) [1–3], with some evidence of reduced work disability in recent years [4–7]. However, work disability remains a considerable problem for many patients with RA [7]. In a large multinational epidemiological study, the probability of continuing to work 2 years after the initial diagnosis was 80 %, falling to 68 % at 5 years [8]. Work disability in RA tends to occur early in the clinical course of the disease [9–11]. The high economic impact on patients, their caregivers and society arising from RA-related work disability [12–14] has prompted the suggestion that the current aim of ‘treating to target’ defined by clinical measures [15] should be refocused on ‘treating to work’, to include also maintenance of working ability [16].

Work disability in RA appears to be multifactorial [9]. One factor contributing to work disability may be the level of RA symptoms experienced by patients. Symptoms of joint stiffness, pain and swelling commonly occur in the morning [17], probably due to the circadian rhythms of inflammatory cytokine release in the early morning [18], with morning stiffness strongly associated with functional disability [19]. Morning stiffness is commonly experienced by patients with RA, particularly early in the course of the disease, and may still be present in patients apparently in remission or with low disease activity [19, 20].

Some results favour the use of MS duration as an outcome measure [21] and some the severity of it [22, 23], even though the severity and duration of morning stiffness seems to correlate strongly [24]. It is not clear if these two ways of assessing morning stiffness reflect different underlying pathologies as there appear to be some differences in the impact on functional outcomes. For example, analysis of a German database showed that severe morning stiffness early in the course of the disease correlated more closely with the decision made by patients with RA to retire from work than duration of morning stiffness [24].

Different time frames [25, 26], methods [27, 28] and study countries [27, 29] can cause variation between results of different studies. An international comparative study conducted at the same time and utilising same methods could produce data, which shows how much inter-country differences affect the findings when time and method elements are simultaneously controlled.

Previous surveys of patients have demonstrated the impact that impaired morning function has on routine activities [30, 31], as well as quality of life [30, 31]. In this study, we aimed to explore the impact of morning stiffness on the working life of patients with RA, with evaluation of differential effect of duration and severity of the symptom.

## Materials and methods

### Participants

We focussed on participants of working age who regularly experience morning stiffness. Because national registers of patients with varying durations or severities of morning stiffness do not exist, methods used to recruit participants varied by country, but mainly involved contacts provided by primary and secondary care physicians and patient associations or databases. Those with a diagnosis of RA for at least 6 months, who were aged between 18 years and the state retirement age for the country of residence, and usually experienced morning stiffness on at least 3 days a week were invited to complete the survey.

### Questionnaire

After exploratory interviews with patients, a structured questionnaire was developed and translated into local languages for use in 11 European countries (Belgium, Denmark, Finland, France, Germany, Italy, Norway, Poland, Spain, Sweden and UK). The countries were selected to be from different parts of Europe and with varying health care systems. Trained interviewers conducted the survey by telephone, taking approximately 10 min to complete the questionnaire with each respondent.

The questionnaire collected socio-demographic background information including age, sex and working status (e.g. full time employment, retired). Disease and morning stiffness characteristics were also collected: respondents recorded time since RA diagnosis (categories of 6 months to less than 1, 1 year or more but less than 2 years and 2 years or more), the typical severity of morning stiffness (assessed on a scale of 0 = no morning stiffness to 10 = severe morning stiffness), the typical duration of morning stiffness before they are able to function normally (in hours and minutes) and the impact of RA-related morning stiffness on quality of life (assessed on a scale of 0 = no impact to 10 = huge impact).

A number of questions were asked to evaluate the impact, if any, of RA-related morning stiffness on working ability or household tasks. All participants were asked the level of agreement (on a scale of 1 = completely disagree to 5 = completely agree) that RA-related morning stiffness resulted in reduced ability to carry out tasks at work or home and also that morning stiffness limited the types of tasks at work or home they were able to carry out. Respondents who were working, wanted to work (i.e. unemployed) or studying were asked if RA-related morning stiffness had ever resulted in inability to work, need to reduce working hours, work flexible hours, work from home, change profession, slowed career progression or had none of these effects. Respondents who were currently working or attending a place of study were asked if RA-related morning stiffness adversely affected their work or study performance at any time in an average week, ever resulted in late arrival at work (and if so, why) or resulted in need for sick leave during the last month.

The contribution, if any, of RA-related morning stiffness to withdrawal from the workforce was evaluated in questions to participants with working status given as retired or partially retired. These respondents were asked if they were not in full time paid work as a direct result of RA and if they took early retirement. Respondents who retired early were asked for factors that influenced their decision. Retired and partially retired respondents were also asked the level of agreement (on a scale of 1 = completely disagree to 5 = completely agree) that morning stiffness due to RA specifically lowered the age at which they retired and also that their career was shorter than they would have liked as a direct result of RA-related morning stiffness.

### Analysis

The responses to questions were recorded for the total group and for subgroups defined by severity and by duration of morning stiffness. Typical severity of morning stiffness was categorised as mild (rating of 0–3), moderate (4–6) or severe (7–10). Typical duration before normal functioning due tomorning stiffness was categorised as <1, 1–2 or >2 h. Responses were also analysed by country of respondent.

The response to questions recording the level of agreement to statements were dichotomised to agreement (score of 4 or 5) or no agreement (other scores). Statistical analyses of the data were based on chi-square tests and Fisher’s exact tests for proportions and Student’s *t* tests. Linear regression models were fitted to test the effects of patient characteristics on duration and severity of morning stiffness, while having the effects of other characteristics simultaneously controlled.

## Results

### Respondents

A total of 1,061 patients were interviewed, approximately 100 from each country included in the survey, with the exception of Norway (55 respondents). Demographic, disease and work status characteristics of the respondents are summarised in Table 1. Both longer duration and greater severity of morning stiffness had significant negative impact on patients’ quality of life (Table 2). Patients’ age and gender did not have independent effects on perceived duration or severity of morning stiffness. However, those who had rheumatoid arthritis longer experienced more severe morning stiffness (*p* < 0.001).

**Table 1:** Demographic, disease and work status characteristics of respondents

	Total	Belgium	Denmark	Finland	France	Germany	Italy	Norway	Poland	Spain	Sweden	UK
Total, *N*	1,061	100	100	103	100	100	100	55	101	102	100	100
Males (%)	22	19	18	13	25	26	25	26	25	26	18	25
Mean age (years)	50	52	49	51	50	50	49	57	49	50	52	51
RA duration >2 years (%)	84	90	92	98	84	85	52	98	72	73	93	96
*Current work status* (%)												
Employed^a^	46	40	47	30	50	46	57	38	53	55	42	47
Retired^b^	21	23	27	24	22	13	8	25	33	13	13	34
Unemployed	5	9	5	3	3	5	3	–	2	6	3	9
Sick leave from work	4	9	3	1	6	11	–	–	–	2	4	3
Student	2	–	5	5	1	1	3	–	5	2	2	–
Homemaker/other	22	19	13	35	18	24	29	34	8	23	36	7
Mean duration of morning stiffness (min)	82	70	82	98	47	98	52	140	59	85	101	88
*Duration of morning stiffness* (%)												
<1 h	33	46	25	19	56	17	51	25	46	18	30	26
1–2 h	30	16	46	26	29	34	20	38	32	27	21	42
>2 h	23	21	29	36	15	19	8	35	11	28	40	32
Varies	14	17	–	19	–	30	21	2	11	27	9	–
Mean morning stiffness severity^c^	5.7	5.6	6.3	5.4	5.9	5.3	6.4	6.0	5.1	5.3	5.7	6.3
*Morning stiffness severity* (%)												
Mild (score 0–3)	15	18	5	18	13	20	6	7	22	24	15	8
Moderate (score 4–6)	48	41	52	46	47	54	40	51	59	50	52	42
Severe (score 7–10)	37	41	43	36	40	26	54	42	19	26	33	50
Mean quality of life^d^	5.9	5.2	5.7	5.9	6.8	5.5	6.3	6.0	5.5	5.3	5.8	7.0

^a^Employed respondents includes those in full time paid work, part time paid work and self-employment

^b^Retired respondents includes those fully and partially retired

^c^Score on scale of 0–10

^d^Impact of RA-related morning stiffness on quality of life

**Table 2:** Relationship between severity and duration of morning stiffness, and impact of morning stiffness on quality of life

	Severity of morning stiffness			*p* value (all comparisons)
	Mild	Moderate	Severe	
Mean duration of morning stiffness (min)	48	69	108	<0.05
Impact of morning stiffness on quality of life (mean score)	3.5	5.5	7.5	<0.05

	Duration of morning stiffness			*p* value (all comparisons)
	<1 h	1–2 h	>2 h	
Severity of morning stiffness (mean score)	5.1	6.0	6.6	<0.05
Impact of morning stiffness on quality of life (mean score)	5.1	6.3	6.9	<0.05

### Impact of RA-related morning stiffness on ability to work

**Fig. 1:**
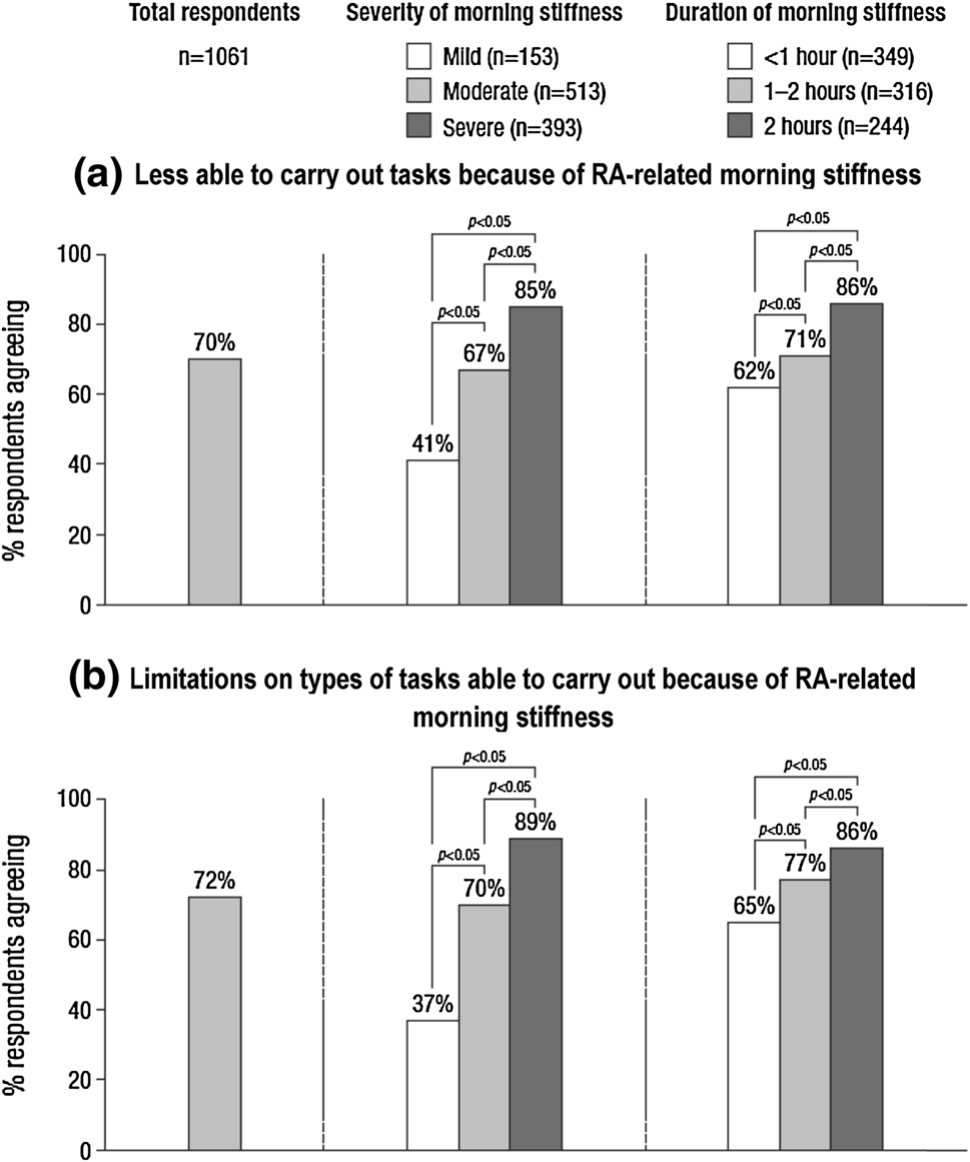
Impact of RA-related morning stiffness on working ability (base: all respondents)

Rheumatoid arthritis-related morning stiffness reduced working ability, with the impact significantly increasing with severity and duration of morning stiffness (Fig. 1). The proportion of respondents stating that they were less able to carry out tasks (at work and home) because of RA-related morning stiffness ranged from 55 % in Sweden to 91 % in Poland. The lowest proportion (55 %) of respondents experiencing limitations on the types of tasks they could carry out due to RA-related morning stiffness was in Sweden, with respondents in France, Norway and Poland showing the highest proportion (85 %).

**Fig. 2:**
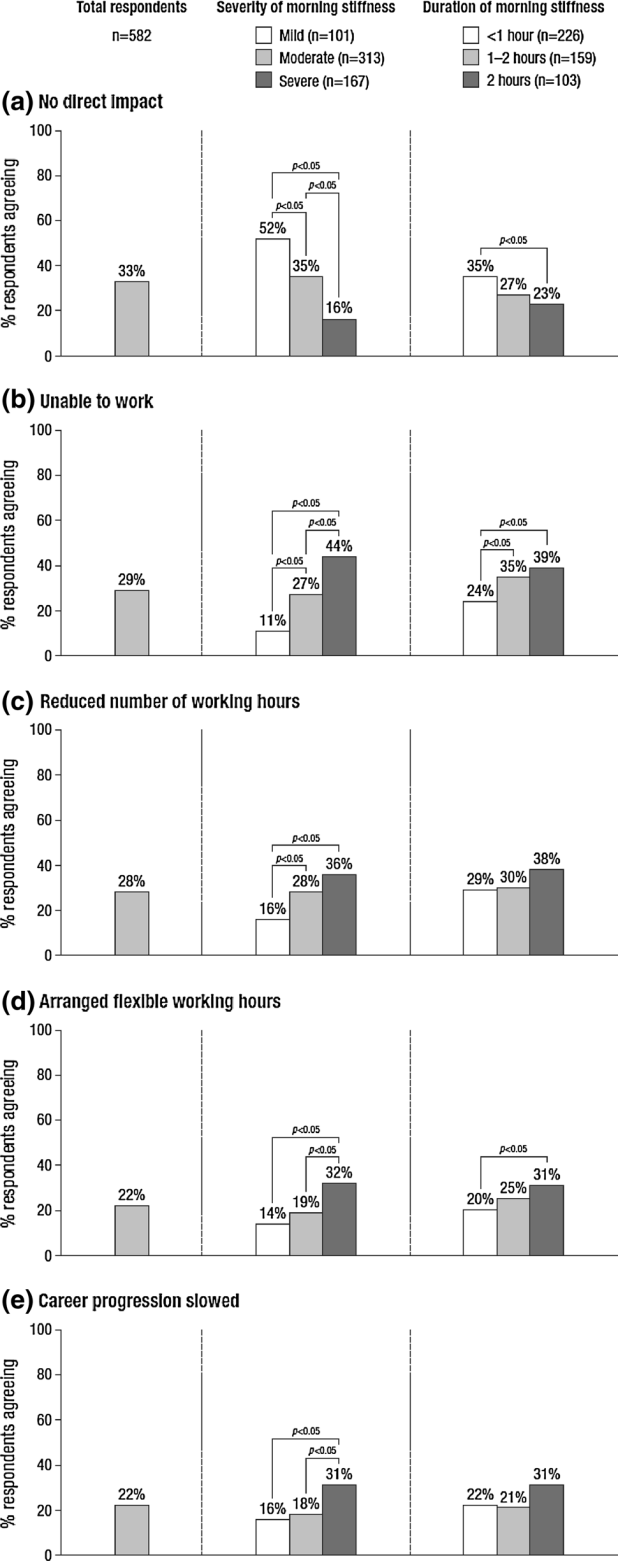
Impact of RA-related morning stiffness on working life (base: respondents who are currently working or attending a place of study or could be working)

Only 33 % of the 582 participants who worked or could be working reported no direct impact of RA-related morning stiffness on their work and career, ranging from 14 % in Norway to 55 % in Poland. Inability to work was reported by 29 % (ranging from 8 % in Spain to 48 % in Norway) and 28 % reported reducing the number of working hours because of RA-related morning stiffness (ranging from 12 % in Poland to 43 % in Norway). The impact appeared to increase with severity of the RA-related morning stiffness, though the relationship with duration of the symptom appeared less pronounced (Fig. 2).

**Fig. 3:**
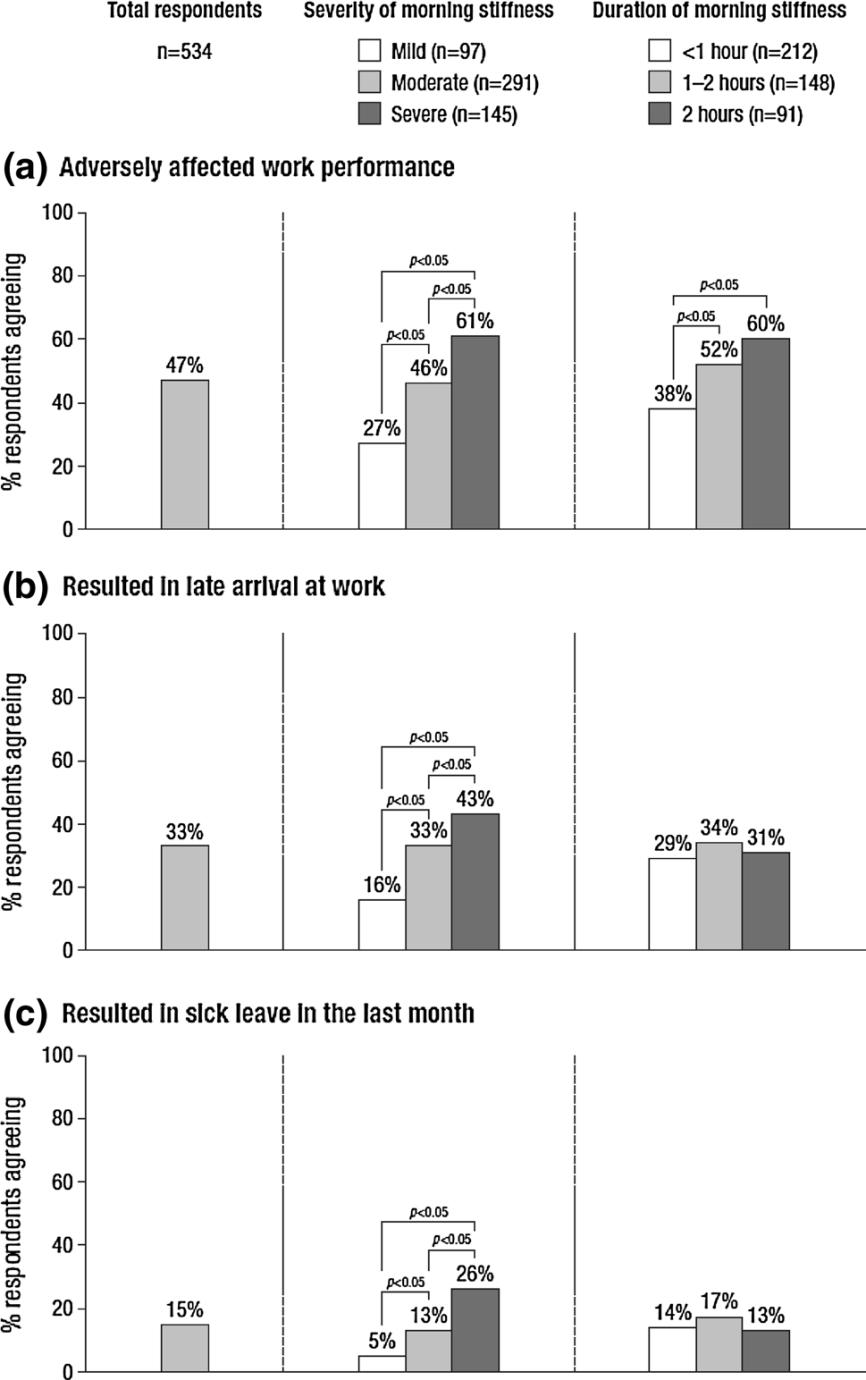
Impact of RA-related morning stiffness on work performance, late arrival at work and sick leave (base: respondents who are currently working/attending a place of study)

Among the 534 participants currently working or attending a place of study, 47 % reported that RA-related morning stiffness adversely affected their performance at work during an average week (range 28 % in Denmark to 67 % in Italy), most commonly because they are less able to perform daily tasks (57 %) and they take more time to do their work (57 %). Late arrival at work because of RA-related morning stiffness was reported by 33 % (ranging from 5 % in Norway to 56 % in Italy), most commonly because of taking longer to get ready (77 %), difficulties driving (19 %) and difficulties using public transport (16 %). A total of 15 % of respondents reported taking sick leave because of RA-related morning stiffness during the previous month (ranging from 2 % in Denmark and Poland to 38 % in France and Italy). The impact of RA-related morning stiffness on work performance, late arrival at work and sick leave appeared to increase with severity, but not necessarily with duration of the symptom (Fig. 3).

### Impact of RA-related morning stiffness on retirement from work

Rheumatoid arthritis-related morning stiffness influenced the decision to take early retirement from work. The majority (71 %) of the 224 retired/semi-retired respondents stopped working earlier than their expected retirement age (ranging from 48 % in Poland to 92 % in Germany). The proportion of respondents retiring early increased with increasing duration of morning stiffness (<1 h: 63 %; 1–2 h: 71 %; >2 h: 88 %; *p* < 0.05 for comparison between >2 h and other groups), but not with increasing severity (mild: 74 %; moderate: 67 %; severe: 74 %).

When asked about factors affecting the decision to retire early from work, 64 % gave RA-related morning symptoms (stiffness) as the reason, with 26 % citing other health issues. RA-related morning stiffness lowered the retirement age for 56 % of retired respondents, and 52 % noted that their career was shorter than they would have liked because of RA-related morning stiffness.

## Discussion

The results of this large pan-European survey highlight the adverse impact of morning stiffness on working life among patients with RA who experience this common symptom. The majority of working patients in this population reported that RA-related morning stiffness affected working ability. RA-related morning stiffness commonly resulted in reduced work performance, late arrival at work and sick leave from work. Among those no longer in paid employment, morning stiffness contributed to early retirement from work. Subgroup analysis suggests that severity and duration of morning stiffness have different effects on working lives of patients with RA. The negative influence of RA-related morning stiffness on overall performance was also demonstrated by the relationship between morning symptoms and quality of life.

The survey found a direct impact of RA-related morning stiffness on working life in two-thirds of the patients included in the survey. This is similar to 74 % of respondents in paid employment in a previous survey reporting a significant impact of impaired morning function on their job [31]. In another study (in an unselected RA population with early RA), 31 % reported diminished capacity in functions required at work [32]. It is perhaps not surprising that morning stiffness, which impairs the ability to get ready in the morning [31], may cause late arrival at work and reduced job performance.

The findings of the survey suggest that RA-related morning stiffness, by adversely impacting performance when at work (presenteeism) and attendance at work (absenteeism), may contribute to the considerable societal costs of RA. Presenteeism has an economic cost that contributes to the cost of lost productivity arising from RA [27]. The survey also showed that RA-related morning stiffness has a direct impact on absenteeism from the work force, due to the need for sick leave, and ultimately, early retirement from work. Both factors are major contributors to the societal cost of RA [13].

The analysis of subgroups defined by severity and duration of morning stiffness revealed subtle distinctions in the impact of RA-related morning stiffness on working life. With respect to current working life, there appeared to be a more pronounced association between severity of the symptom than duration. In contrast, early retirement in those no longer in work appeared to have a closer association with duration of morning stiffness than severity, though current symptoms may not reflect those at the time of retirement. There is likely to be some relationship between severity and duration of morning stiffness, as indicated by the increasing severity as duration increased and vice versa. A similar relationship has been noted previously in both a survey of patients and a database analysis [24, 31]. Both of these earlier studies noted a significant association between severity of morning stiffness and impact on ability to work, but a weaker association with duration of the symptom. It was noted many years ago that the assessment of severity has better measurement characteristics than duration of morning stiffness [22]. However, the differential impact of morning stiffness duration and severity is unlikely to be due to how information on duration was solicited or measured as this was consistent for each individual included in the survey.

Our findings corroborate earlier studies showing that apart from having an impact on work ability, morning stiffness seems to be related to overall quality of life [30, 31]. Despite improvements in medical treatment of RA, in particular the availability of biologics, morning stiffness can sometimes remain insufficiently managed and then deserve more attention in patient evaluation and treatment of RA.

A strength of this survey was the large size and the wide distribution of European countries involved. The numbers of patients per country were estimated to be sufficient for comparisons between countries. By asking the role of morning stiffness in perceived disadvantage in each aspect of working life (i.e. absenteeism, presenteeism, late arrivals and early retirement), the attempt was to identify the independent role of morning stiffness and exclude possible confounding effects of other symptoms and other concomitant diseases. Findings for the impact of RA-related morning stiffness within subgroups of respondents defined by country showed marked differences. This variation may affect the impact of RA-related morning stiffness on productivity. The variation in RA-related burden of illness, quality of life and work disability between countries has been reviewed [5, 14]. Our study use similar methods and study population in 11 countries and found results comparable to average European values in these reviews.

The study has some limitations. In common with other patient surveys, interpretation of the descriptive findings may be affected by confounding factors. One potential confounder of interest is respondent age. However, the pattern of response across subgroups defined by age was different to that defined by severity and duration of morning stiffness, suggesting that the impact of morning stiffness may not be strongly dependent on age.

Another limitation of the survey was the focus on a selected population of patients of working age who experienced regular morning stiffness. Given the specific interest in the impact of morning stiffness on working life, this was a pragmatic design decision. With the peak incidence for development of RA being 30–60 years of age [33] and the widespread occurrence of morning stiffness [19, 20], this approach may not have produced any notable selection bias. However, the use of a selected population means that the findings from this survey cannot be extrapolated to an unselected population of patients with RA. Because the recruitment strategies varied slightly by country, this introduces a possibility for selection bias. Also, the recruitment procedure did not necessarily include confirmation of the diagnosis of RA by a rheumatologist; thus, it is possible that some non-RA patients may have been included in some subsamples. The patients’ medical treatments were not solicited, because this study focused on effects of morning stiffness on work.

In conclusion, this large survey conducted in 11 European countries has shown that morning stiffness reduces ability to work and contributes to early retirement. Impairment appears to be affected by both severity and duration of morning stiffness. Morning stiffness seemed to be an important symptom of rheumatoid arthritis when considering the effects of the disease on various aspects of working life. Further studies on its independent role are needed.
